# Cross-Sectional Associations of Smoking and E-cigarette Use with Self-Reported Diagnosed Hypertension: Findings from Wave 3 of the Population Assessment of Tobacco and Health Study

**DOI:** 10.3390/toxics9030052

**Published:** 2021-03-09

**Authors:** Connor R. Miller, Hangchuan Shi, Dongmei Li, Maciej L. Goniewicz

**Affiliations:** 1 Roswell Park Comprehensive Cancer Center, Department of Health Behavior, Buffalo, NY 14263, USA; maciej.goniewicz@roswellpark.org; 2 Department of Clinical & Translational Research, University of Rochester Medical Center, Rochester, NY 14627, USA; hangchuan_shi@urmc.rochester.edu (H.S.); dongmei_li@urmc.rochester.edu (D.L.); 3 Department of Public Health Sciences, University of Rochester Medical Center, Rochester, NY 14627, USA

**Keywords:** tobacco, e-cigarettes, smoking, hypertension, epidemiology

## Abstract

Following their introduction a decade ago, electronic cigarettes (e-cigarettes) have grown in popularity. Given their novelty, knowledge of the health consequences of e-cigarette use remains limited. Epidemiologic studies have not comprehensively explored associations between e-cigarette use and hypertension, a highly prevalent health condition and major contributor to cardiovascular disease burden. In this study, cross-sectional associations of cigarette smoking and e-cigarette use (vaping) with self-reported diagnosed hypertension were evaluated among 19,147 18–55 year old respondents in Wave 3 (2015–2016) of the Population Assessment of Tobacco and Health Study. Multivariable analyses first modeled smoking and vaping as separate 2-category variables, then as a 6-category composite variable accounting for former smoking. After adjusting for potential confounders, current vaping (aOR = 1.31; 95%CI: 1.05–1.63) and current smoking (aOR = 1.27; 95%CI: 1.10–1.47) were both associated with higher odds of hypertension. In analyses modeling smoking and vaping compositely, respondents who were concurrently smoking and vaping had the highest odds of hypertension (aOR = 1.77; 95%CI: 1.32–2.39 [referent: never smokers]). These results differ somewhat from prior epidemiologic studies of vaping and respiratory outcomes, which consistently report smaller point estimates for current vaping than for current smoking. Our findings reinforce the uncertainty surrounding long-term health consequences of vaping, as well as highlight important distinctions between respiratory and cardiovascular outcomes when considering the harm reduction potential of e-cigarettes.

## 1. Introduction

Tobacco’s status as a leading cause of preventable disease and premature mortality spans many decades [1]. While intense focus from researchers and policy makers has contributed substantially to recent decreases in use [2], tobacco remains a major public health concern: in 2017, an estimated 7.1 million deaths and the loss of 182 million disability-adjusted life years were attributed to tobacco use across the globe [3]. Notably, the vast majority of tobacco-related death and morbidity are caused by smoke from combusted tobacco products [4], which contains numerous cardiovascular toxicants [5]. A seminal 2005 publication estimated that 1 in 10 deaths from cardiovascular disease (CVD) could be attributed to tobacco smoking in the year 2000 [6], reinforcing the importance of smoking as a modifiable risk factor in efforts to reduce global burden of CVD.

In light of the detriment caused by smoking tobacco, electronic cigarettes (e-cigarettes) were developed as alternatives to combusted cigarettes in the late 2000s. E-cigarettes encompass a range of devices which heat and aerosolize a solution that typically contains nicotine and a mixture of propylene glycol, glycerin and various flavoring additives. Laboratory studies have shown that the amount and concentration of toxicants in e-cigarette aerosol are substantially lower than in cigarette smoke [7,8,9]. As such, e-cigarettes are often promoted as potentially-modified risk products compared with cigarettes, and the majority of adult e-cigarette users (vapers) are current or former cigarette smokers, many of whom reference ‘quitting smoking’ as a primary reason for initiating use [10].

The potential public health implications of smokers fully transitioning away from cigarettes in favor of vaping are still not well understood. Knowledge regarding associations between vaping and a multitude of clinical health outcomes, including cardiovascular conditions, is currently limited. A handful of published studies have examined cross-sectional associations between CVD and vaping in large free-living samples of the US adult population [11,12,13,14]. Conflicting results have been reported, and the topic has incurred contentious debate, particularly surrounding methodological decisions regarding confounding of the association by history of cigarette smoking among adult e-cigarette users.

Notably, epidemiological studies have yet to examine associations between vaping and important clinical risk factors of CVD (i.e., diabetes mellitus, hypertension, hyperlipidemia), most of which are known to be adversely associated with smoking cigarettes. Of particular interest is hypertension, as it remains the leading risk factor for CVD worldwide, and for which an estimated 10.4 million deaths and 218 million disability-adjusted life years were attributed in 2017 [3]. Transient increases in systolic blood pressure have been observed following an acute bout of vaping in humans [15], while accelerated aortic stiffness and abnormal vascular inflammation have been reported after substantial exposure to e-cigarette aerosols in mice and in vitro studies, respectively [16,17], both of which could contribute to the development of hypertension if confirmed in humans [18]. Likewise, cigarette smoking causes a short-term spike in blood pressure [19], while its adverse impact on endothelial function, vascular injury, and arterial compliance suggest a potential role in hypertension pathogenesis [20]. However, epidemiological studies of associations between smoking and chronic blood pressure alterations have reported mixed findings, with some publications observing a higher and others a lower risk of hypertension among habitual cigarette smokers compared to never smokers [21].

Given the equivocal state of evidence for cigarette smoking and a lack of evidence for vaping, the present study evaluated cross-sectional associations of vaping and cigarette smoking with self-reported hypertension in a nationally representative sample of US adults, with a focus on young and middle-aged adults. Multiple statistical modeling approaches were employed in an attempt to scrutinize the association between vaping and hypertension independently from cigarette smoking, as well as approximating cumulative exposure to both products together.

## 2. Materials and Methods

### 2.1. Study Population

The present study analyzed data from the Wave 3 Population Assessment of Tobacco and Health (PATH) Study public use files (available at: https://www.icpsr.umich.edu/web/NAHDAP/studies/36498/datadocumentation (accessed on 8 March 2021)). The PATH Study is a nationally representative prospective cohort study evaluating tobacco use behaviors, perceptions, and tobacco-related health outcomes among youth and adults in the United States [22]. The PATH Study utilized a four-stage stratified area probability sampling method to assemble the baseline cohort, with a two-phase design for sampling adults at the final stage. Additional information regarding study design and methodology has been published [23].

Initial data collection for Wave 1 occurred between September 2013 and December 2014. The Wave 1 weighted recruitment rate was 54.0%, of which 74.0% completed the survey, resulting in a baseline cohort containing 32,320 adult respondents (age 18+ years). Subsequent waves of data were collected from October 2014 to October 2015 (Wave 2; n = 28,362 adults) and again from October 2015 to October 2016 (Wave 3; n = 28,148 adults). The weighted response rates at Wave 2 and Wave 3 were 83.2% and 78.4%, respectively.

Compared to older adults, (a) hypertension is less widespread and (b) survival bias and reverse causality are less likely to influence associations of interest in this study. Therefore, our primary analyses focused on young and middle-aged adults (18–54 years) at Wave 3 (n excluded for being 55+ years = 6095). In primary analyses, we further excluded respondents who were current-established users (i.e., had ever used a specified product fairly regularly and currently use every day or some days) of ‘other’ tobacco products: traditional cigars, hookah, cigarillos, filtered cigars, pipes, snus, or smokeless tobacco (additional n excluded = 2906). This left an analytic sample of n = 19,147.

### 2.2. Assessment of Hypertension

The outcome of interest for this analysis was self-reported diagnosed hypertension. At Wave 1, PATH respondents were asked “Has a doctor, nurse or other health professional ever told you that you had high blood pressure?”, and at Waves 2 and 3 they were asked “In the past 12 months, has a doctor, nurse or other health professional told you that you had high blood pressure?”. Wave 3 respondents who reported ever being diagnosed with high blood pressure were subsequently asked “In the past 12 months, has your high blood pressure been under control?”. Those who selected *yes* or *no* to this question were classified as having hypertension, while those who selected *never had high blood pressure* were re-classified as not having hypertension (See Figure S1 for additional details).

### 2.3. Assessment of Smoking and Vaping Status

Separate binary variables were defined for current smoking and current vaping. Current vapers had ever used e-cigarettes, ever used them fairly regularly, and currently used them every day or some days. Current smokers had smoked at least 100 cigarettes in a lifetime, and currently smoked every day or some days. Furthermore, integrating the category of former smokers (smoked 100 cigarettes in their lifetime, but did not currently smoke on an everyday or someday basis at time of survey), we derived a composite smoking and vaping variable with six categories: (1) exclusive vapers who were never smokers, (2) exclusive vapers who were former smokers, (3) dual users, (4) exclusive smokers, (5) former smokers and (6) never smokers.

### 2.4. Assessment of Covariates

To control for potential confounding, the following variables were adjusted for in all primary multivariable analyses: age, sex, race-ethnicity, education, annual household income, insurance status, marital status, leisure-time physical activity, body mass index (BMI), heavy alcohol use, hypercholesterolemia, and diabetes mellitus. Leisure-time physical activity categories were defined according to the question “in a typical week, how many days do you do any physical activity or exercise of at least moderate intensity, such as brisk walking, bicycling or swimming at a regular pace?” (0 days/week, 1–3 days/week, ≥4 days/week). Heavy alcohol use was defined as having 5 or more alcoholic drinks in one day on 5 or more days in the past month. Hypercholesterolemia and diabetes mellitus were classified according to similar case-finding questions as the outcome variable (hypercholesterolemia: [a] “[Have you ever been told by/ In the past 12 months, has]…a doctor, nurse or other health professional told you that you had high cholesterol?”, [b] “In the past 12 months, have you taken any medications to reduce cholesterol?”; diabetes: [a] “[Have you ever been told by/ In the past 12 months, has]…a doctor, nurse or other health professional that you have diabetes, sugar diabetes, high blood sugar, or borderline diabetes?” [b] “What type of diabetes do you have?”). All adjusted covariates were employed as categorical variables.

### 2.5. Statistical Analysis

Analyses were conducted using survey analysis procedures (i.e., *proc surveylogistic*) in SAS v9.4 (SAS Institute Inc., Cary, NC, USA), with a significance level for two-sided tests set at 0.05. The balanced repeated replication (BRR) method was used to form replicate weights in variance estimation to account for the complex sampling design in the cross-sectional PATH Wave 3 data. We used weighted frequency distributions and the Rao-Scott modified likelihood ratio test to examine bivariate associations between covariates and current vaping and smoking status. Multivariable weighted logistic regression models estimated adjusted odds ratios (aORs) and 95% confidence intervals (CIs) for associations of vaping and smoking with hypertension. Two sets of primary multivariable analyses were conducted: first, modeling smoking and vaping as separate 2-category variables, then modeling smoking and vaping as a 6-category composite variable. 

In sensitivity analyses (see supplement files), current users of ‘other’ tobacco products were re-introduced to the analytic sample. Two additional binary covariates controlling for current combusted (traditional cigars, hookah, cigarillos, filtered cigars, or pipes) and smokeless (snus or smokeless) tobacco use were incorporated into these multivariable logistic regression models. Missing data were handled as listwise deletions in multivariable models (details provided in Tables S2 and S3).

## 3. Results

Among the 19,147 PATH Wave 3 respondents included in the analytic sample, there were 1100 (3.7% [3.4–4.0]) current vapers and 5654 (19.5% [18.7–20.3]) current smokers (Table 1). Most current vapers were current or former smokers. Aside from insurance status, history of hypercholesterolemia, and history of diabetes mellitus, all other Table 1 characteristics were significantly associated with current vaping status (χ^2^ *p* < 0.05). All Table 1 characteristics except BMI and history of diabetes mellitus were significantly associated with current smoking status (χ^2^ *p* < 0.05). Table S1 shows descriptive statistics according to the six-category composite smoking and vaping variable. Over three-quarters of current exclusive vapers who never smoked were 18–24 years old, and almost half had a BMI <25 kg/m^2^. The four categories comprised of current or former smokers were predominantly 35 years and older, while a clear majority had BMI >25 kg/m^2^.

**Figure 1:**
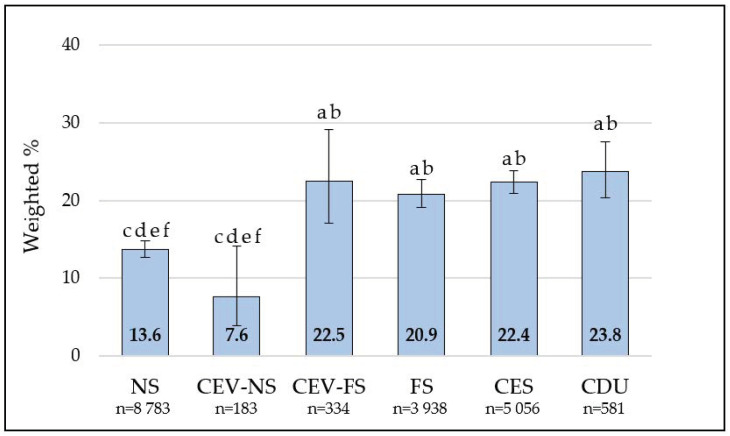
Prevalence of self-reported hypertension according to smoking and vaping status. NS = never smoker; CEV-NS = current exclusive vaper who never smoked; CEV-FS = current exclusive vaper who formerly smoked; FS = former smoker; CES = current exclusive smoker; CDU = current dual user. Superscript letters indicate significant differences during Bonferroni-adjusted pairwise comparisons: a = NS; b = CEV-NS; c = CEV-FS; d = FS; e = CES; f = CDU.

Overall, 17.3% (16.4–18.1) of respondents had self-reported hypertension in 2015–2016 (Table 1). Self-reported hypertension was higher among current vapers than those who were not current vapers, as well as among current smokers than those who were not current smokers. The prevalence of self-reported hypertension across composite smoking and vaping categories are presented in Figure 1. In pairwise comparisons, prevalence among never smokers and current exclusive vapers who had never smoked each differed from the other four categories.

Table 2 displays multivariable results for weighted logistic regression modeling smoking and vaping as separate risk factors. Following adjustment for relevant sociodemographic factors, health behaviors, and clinical variables, current smokers had 27% higher odds of hypertension than those who were not, and current vapers had 31% higher odds of hypertension than those who were not. Relationships between established risk factors for hypertension were as expected, with particularly strong associations seen for age, BMI, hyperlipidemia and diabetes mellitus.

**Figure 2:**
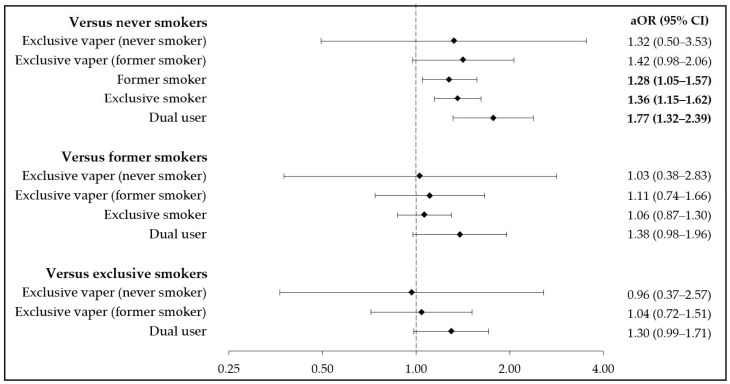
Multivariable odds for hypertension among the analytic sample, modeling smoking and vaping as a composite variable. All results come from weighted logistic regression models controlling for age, race-ethnicity, sex, annual household income, education, leisure-time physical activity, BMI, heavy alcohol use, insurance status, marital status, hypercholesterolemia, and diabetes mellitus. aOR = adjusted odds ratio; CI = confidence interval.

Multivariable results from modeling smoking and vaping as a composite variable are shown in Figure 2. Former smokers, current exclusive smokers, and current dual users had 28%, 36%, and 77% higher odds of hypertension than never smokers, respectively. No significant differences were observed in the odds of hypertension with former smokers or current exclusive smokers as the referent group. Point estimates in Figure 2 analyses were generally higher for current vapers who formerly smoked than for those who never smoked, but no statistically significant findings were observed.

## 4. Discussion

In this nationally representative cross-sectional study of US adults aged 18–54 years, both current vaping and current smoking were significantly associated with self-reported diagnosed hypertension when modeled as separate parameters and controlling for potential confounding variables. The strength of association was similarly modest for both factors: current vapers had 31% higher odds of hypertension than those who did not currently vape, while current smokers had 27% higher odds of hypertension than those who did not currently smoke. Additionally, when modeling a combined smoking and vaping variable that accounted for former smoking, current dual users had 77% higher odds of hypertension, while current exclusive smokers had 36% higher odds. Although the small number of vapers in the analytic sample limited statistical power upon additional stratification, the association between current exclusive vaping and hypertension appeared slightly stronger among those who formerly smoked.

This was the first epidemiologic study to comprehensively evaluate the association between vaping and hypertension in an adult sample. Vaping is most common among three distinct groups of individuals: (a) youth and young adults, many of whom have never been habitual cigarette smokers, (b) adult current smokers who are interested in quitting, and (c) adult former smokers who have successfully quit smoking but continue vaping in place of cigarettes [24,25]. Whereas adult current and former smokers are often interested in using e-cigarettes to reduce harmful effects of smoking [26], this is clearly not the case for tobacco-naïve vapers. Therefore, it is important to evaluate potential harms of vaping on both an absolute level as well as relative to smoking cigarettes. Under the assumption that these cross-sectional results can be extrapolated to approximate risk estimates, our findings would suggest both vaping and smoking have a similar detrimental influence on blood pressure regulation, while utility of e-cigarettes for harm reduction in smokers may be limited with respect to hypertension. However, this interpretation should be met with scrutiny; even the most adept cross-sectional studies are ill-suited for causal inference. Specific to this study, data from earlier waves of the PATH Study have indicated that e-cigarette use is frequently a transient phase [27], whereas cigarette smoking is more persistent. Combined with the recency of e-cigarettes in the marketplace, differential levels in cumulative exposure to the two products is important to note when interpreting prevalence odds ratios [28]. Furthermore, even when attempting to control for former smoking in our models, some level of residual confounding is likely among current exclusive vapers due to varying levels of lifetime exposure to cigarette smoking (e.g., some current exclusive vapers had smoked ‘a pack a day’ for 20 years and just recently quit, others had quit smoking 5 years ago). The possibility of reverse causality playing a role in our results also cannot be dismissed, as smokers with prevalent hypertension might be more interested in switching to vaping than someone who has not been diagnosed with hypertension.

Even with these limitations in mind, our findings are concerning from a public health perspective. Hypertension is a relatively common condition [29] and plays a causative role in the pathogenesis of atherosclerosis [30]. As such, it remains a leading cause of disease burden worldwide [3,31], and even minor differences in disease risk would have significant ramifications at the population level [32]. That current dual users had the highest odds of hypertension is unsurprising, given previous research indicating higher rather than lower exposure to toxicants among current dual users compared with current exclusive smokers [33]. Our results comparing current vaping with current smoking are especially interesting, as previous cross-sectional studies have also observed similar point estimates for current smoking and current vaping when evaluating associations with major adverse cardiovascular endpoints (e.g., stroke, myocardial infarction) [11,13,14]. This contrasts with cross-sectional studies of respiratory outcomes (e.g., asthma, COPD, chronic wheezing), which have reported relatively smaller point estimates for current vaping than for current smoking [34,35,36]. Two recent studies have also examined inflammatory biomarkers relevant to cardiovascular health, observing similar levels among exclusive vapers and non-tobacco users while exclusive smokers and dual users had higher levels [37,38]. Additionally, while our analysis evaluated hypertension solely as an outcome variable, the potential for vaping to act synergistically with blood pressure in influencing overall cardiovascular risk is plausible and important to consider in future studies. This has been the prevailing hypothesis for the relationship between smoking and blood pressure for some time [39,40,41].

### 4.1. Previous Research on Vaping and Blood Pressure

#### 4.1.1. Absolute Harms

Given the recency of e-cigarettes, the long-term health effects of vaping remain unclear. Thus far, evidence supporting a hypothetical role for vaping in hypertension development primarily stems from animal models. A handful of mechanisms have been established in studies of mice regularly exposed to high levels of e-cigarette emissions. These include overactivation of the renin–angiotensin–aldosterone system via vaping-induced increases in circulating levels of inflammatory cytokines [42] and heightened aortic pulse wave velocity via vaping-induced increases in aortic stiffness [16]. Additionally, human experimental studies have evaluated the acute blood pressure response to vaping in small samples of adult current vapers. A recent meta-analysis of these studies reported mean increases of 2.0 mmHg for both systolic blood pressure and diastolic blood pressure following vaping sessions of 5–30 minutes in duration [15]. Only one of the studies included in the meta-analysis reported on longevity of vaping-induced blood pressure elevations: following a five minute vaping session, systolic blood pressure returned to baseline levels after an average of 25 minutes at resting (diastolic blood pressure was not measured) [43]. With regard to the chronic blood pressure adaptations which characterize hypertension, the applicability of these studies is uncertain; similar acute blood pressure responses are observed for other exposures including physical activity, which is known to be protective against hypertension development [44,45].

#### 4.1.2. Relative Harms

While knowledge of the absolute harms of e-cigarettes remains limited, there have been some randomized controlled trials (RCTs) that provide valuable insight towards the relative harms of vaping compared to smoking, including two that explored blood pressure as a secondary outcome. The ECLAT study was a 1-year smoking cessation trial where adult smokers were given one of three e-cigarettes of varying nicotine concentrations with the aim of quitting smoking. Among respondents who fully abstained from smoking from week 12 to the end of follow up (n = 18), no significant changes in systolic blood pressure were observed for baseline normotensive participants (systolic blood pressure < 130 mmHg and diastolic blood pressure < 85 mmHg), while those with baseline elevated blood pressure saw an average 16.3 (standard deviation: ±11.3) mmHg reduction in systolic blood pressure [46]. In the more recent VESUVIUS trial, adult smokers were randomized to a control group (continued smoking; n = 47) or one of two e-cigarette intervention arms, one group transitioning to nicotine-containing e-cigarettes (n = 37) and the other to nicotine-free e-cigarettes (n = 37). After a 4-week follow up, the mean change in systolic blood pressure differed significantly across the 3 arms: continued smokers saw a reduction of 1.9 mmHg, while the nicotine-containing intervention arm saw a reduction of 4.2 mmHg and the nicotine-free intervention arm saw a reduction of 9.7 mmHg [47]. Notably, both trials enrolled participants who smoked an average of 18–20 cigarettes daily, and the nicotine-containing e-cigarette intervention arms utilized early generation products with nicotine concentrations of 5.4–16.0 mg/ml. Given the rising popularity of e-cigarettes with substantially higher nicotine concentrations (e.g., 56 mg/ml) [48], future RCTs evaluating these newer products as well as studies that enroll less frequent smokers will expand understanding of potential cardiovascular harm reduction.

In addition to the aforementioned RCTs which have considered blood pressure as a secondary outcome, Polosa et al. explored blood pressure changes in a 2016 prospective analysis of 89 baseline hypertensive smokers (43 e-cigarette adopters, 46 continued exclusive smokers) [49]. 20 of the e-cigarette adopters abstained from cigarettes completely during follow-up, while the other 23 decreased their daily cigarette consumption from 20 to 5 cigarettes on average. After a 12-month follow-up, the 43 participants that adopted regular e-cigarette use saw respective decreases of 10 mmHg for systolic blood pressure and 6 mmHg for diastolic blood pressure. Stratified analyses indicated meaningful blood pressure reductions were possible for those e-cigarette adopters who reduced rather than quit smoking cigarettes, albeit to a lesser degree than those who fully abstained from cigarette smoking.

### 4.2. Strengths and Limitations

In addition to being the first epidemiologic study of vaping and hypertension, our study has a handful of strengths. The PATH Study is an exceptionally comprehensive data source with respect to tobacco use, even including biomarker data for a large subset of participants. This affords a unique opportunity to validate self-reported tobacco use measures that other epidemiologic datasets may not have. Indeed, prior assessments of specificity and sensitivity for self-reported smoking and vaping status instill confidence that exposure misclassification is not a major concern in our analysis [50]. PATH’s detailed tobacco information also makes it ideal for studying novel products and accounting for use of multiple products. In order to examine the “pure” associations of vaping and smoking with hypertension, we used two strategies to mitigate the potential confounding influence of other tobacco products: (a) primary models excluded users of tobacco products other than cigarettes or e-cigarettes; (b) in sensitivity analyses, we re-introduced the ‘other’ tobacco product users and adjusted for other tobacco products as potential confounders (Figure S2; results were consistent with those from the Figure 2 analyses). We also excluded people aged 55+ years to avoid survivorship bias, as the current research paradigm indicates that smoking and hypertension have combined effects on risk of cardiovascular disease mortality [39,40,41]. This would disproportionately influence survival among middle and older age smokers, introducing a bias among the 55+ age group. This phenomenon was likely observed in supplemental analyses of Wave 3 PATH respondents that were 55+ years old, for whom the observed association with hypertension was null for current smoking and inverse for current vaping (Table S4).

Along with previously mentioned limitations, a constraint of PATH is the lack of dietary information, which is particularly relevant for hypertension (e.g., sodium and potassium intake) [51]. As health behaviors tend to cluster [52], smokers may have been more likely to maintain nutrition habits associated with higher risk of developing hypertension, as previously reported in other studies [53]. While we are unaware of publications assessing the relationship directly, this could also be true for vapers, the majority of which are current or former smokers. Regarding the outcome of interest, self-reported diagnosed hypertension has not yet been validated within the PATH Study. However, similar hypertension case-finding questions have demonstrated reproducibility and substantial agreement with clinical blood pressure measurements in other representative samples of the US adult population [54,55]. Furthermore, the prevalence of self-reported hypertension at Wave 3 of the PATH Study was consistent with those reported by the Centers for Disease Control and Prevention in 2015–2016 [29]. It is also important to consider a potential detection bias due to the outcome variable’s reliance on physician diagnosis of hypertension. Blood pressure measurement is a common screening procedure across a broad set of health professional visits (e.g., annual physicals, emergency room visits, etc.). This means those who see a physician more frequently could have more opportunities to be diagnosed with hypertension, even if their visits are for reasons seemingly unrelated to high blood pressure. As it pertains to our study, it is possible that tobacco users and non-users differ in their frequency of health professional visits. We conducted a sensitivity analysis looking at the proportion of each tobacco user group who selected “Yes” to the question “In the past 12 months, have you seen a medical doctor?” at Wave 3 of PATH, as well as at the two previous waves for our study’s analytic sample (Table S5). There were statistically significant differences across the 6 tobacco use categories overall for doctor visitations at each wave (χ^2^ *p* <0.001), however the absolute differences in proportions between groups were for not especially large. Finally, the e-cigarette marketplace has evolved since the time of this survey to include devices of varying power as well as substantial ranges in nicotine concentration [56,57]. It will be important to re-evaluate associations with health outcomes as e-cigarette technology continues to evolve.

## 5. Conclusions

In summary, this cross-sectional analysis of young and middle-age adults in Wave 3 of the PATH Study found a positive albeit weak association between vaping and self-reported hypertension, of similar magnitude to that of cigarette smoking and hypertension. Our findings underscore the importance of more rigorous longitudinal research into the health effects of e-cigarettes, reinforcing the uncertainty surrounding long-term ramifications of vaping. Moreover, the results suggest important distinctions should be made between respiratory and cardiovascular outcomes when considering the harm reduction potential of e-cigarettes.

## Figures and Tables

**Table 1:** Descriptive statistics for the analytic sample overall, stratified by current vaping status, and stratified by current smoking status.

	Overall Sample (n = 19,147)		Current Vaping Status				Current Smoking Status			
Characteristic			No (n = 18,013)		Yes (n = 1100)		No (n = 13,481)		Yes (n = 5654)	
	n	% (95% CI)	n	% (95% CI)	n	% (95% CI)	n	% (95% CI)	n	% (95% CI)
Hypertension *†										
No	16,267	82.7 (81.9–83.6)	15,344	82.9 (82.0–83.7)	897	78.7 (75.8–81.3)	11,851	84.0 (83.0–84.9)	4404	77.4 (76.0–78.8)
Yes	2859	17.3 (16.4–18.1)	2650	17.1 (16.3–18.0)	201	21.3 (18.7–24.2)	1618	16.0 (15.1–17.0)	1241	22.6 (21.2–24.0)
**Vaping status †**										
Never vaper	16,040	91.3 (90.8–91.8)	---	---	---	---	11,968	95.6 (95.2–95.9)	4064	73.8 (72.2–75.4)
Former vaper	1565	5.0 (4.6–5.3)	---	---	---	---	618	2.3 (2.1–2.6)	946	15.9 (14.7–17.1)
Current vaper	1100	3.7 (3.4–4.0)	---	---	---	---	517	2.1 (1.8–2.4)	581	10.3 (9.4–11.3)
Smoking status *										
Never smoker	2832	16.2 (15.3–17.1)	10,227	66.3 (65.0–67.6)	184	12.9 (10.8–15.5)	---	---	---	---
Former smoker	10,426	64.3 (63.0–65.6)	2498	15.6 (14.7–16.5)	333	32.7 (29.4–36.2)	---	---	---	---
Current smoker	5654	19.5 (18.7–20.3)	5056	18.1 (17.4–18.9)	581	54.4 (50.4–58.2)	---	---	---	---
Age *†										
18–24 years	7238	18.7 (18.2–19.3)	6838	18.5 (17.9–19.1)	389	25.1 (22.5–28.0)	6154	20.2 (19.5–20.9)	1082	12.6 (11.7–13.5)
25–34 years	4985	27.3 (26.4–28.3)	4695	27.3 (26.3–28.3)	282	29.4 (26.6–32.3)	3265	26.6 (25.4–27.8)	1718	30.5 (28.9–32.1)
35–44 years	3549	26.0 (25.0–27.1)	3300	26.0 (25.0–27.1)	239	24.9 (22.3–27.7)	2133	25.5 (24.2–26.7)	1412	28.3 (26.8–29.9)
45–54 years	3375	27.9 (27.0–28.9)	3180	28.2 (27.3–29.1)	190	20.6 (17.9–23.7)	1929	27.7 (26.7–28.8)	1442	28.6 (27.3–30.1)
Sex *†										
Female	10,505	53.8 (53.1–54.5)	9951	54.1 (53.4–54.8)	538	44.9 (41.3–48.5)	7419	54.8 (53.9–55.6)	3081	49.7 (48.2–51.1)
Male	8626	46.2 (45.5–46.9)	8046	45.9 (45.2–46.6)	562	55.1 (51.5–58.7)	6048	45.2 (44.4–46.1)	2571	50.3 (48.9–51.8)
Race-ethnicity *†										
Non-Hispanic White	10,428	59.4 (58.7–60.1)	9627	58.8 (58.1–59.6)	786	76.0 (72.7–79.0)	6792	57.3 (56.4–58.1)	3632	68.7 (67.0–70.3)
Non-Hispanic Black	2674	11.6 (11.1–12.1)	2613	11.8 (11.3–12.4)	57	5.3 (4.0–7.0)	1968	11.6 (11.0–12.2)	703	11.7 (10.7–12.9)
Hispanic	4256	19.8 (19.2–20.4)	4083	20.1 (19.5–20.7)	161	11.5 (9.4–14.0)	3432	21.2 (20.5–22.0)	820	13.6 (12.6–14.6)
Non-Hispanic Other	1580	9.2 (8.6–9.7)	1485	9.3 (8.7–9.8)	94	7.2 (5.4–9.4)	1162	9.9 (9.3–10.6)	417	6.0 (5.3–6.8)
Annual household income *†										
≥USD 50,000	6793	48.3 (47.0–49.5)	6449	48.7 (47.5–50.0)	340	37.1 (33.8–40.6)	5402	52.8 (51.5–54.2)	1388	29.7 (27.8–31.5)
<USD 50,000	11,045	51.7 (50.5–53.0)	10,331	51.3 (50.0–52.5)	689	62.9 (59.4–66.2)	7043	47.2 (45.8–48.5)	3994	70.3 (68.5–72.2)
Education status *†										
Bachelors and beyond	4085	30.9 (30.2–31.5)	3949	31.5 (30.8–32.2)	133	14.4 (11.9–17.3)	3514	35.4 (34.6–36.2)	570	11.8 (10.6–13.3)
Some college	6812	33.0 (32.2–33.8)	6309	32.6 (31.8–33.4)	496	45.5 (42.1–48.9)	4792	32.5 (31.6–33.4)	2019	35.3 (33.8–36.9)
High school or less	8166	36.1 (35.4–36.8)	7681	35.9 (35.2–36.7)	463	40.1 (36.6–43.7)	5122	32.1 (31.2–33.1)	3035	52.8 (51.1–54.5)
Leisure-time physical activity *†										
≥4 days/week	7638	38.2 (37.0–39.3)	7191	38.1 (37.0–39.3)	439	39.1 (35.8–42.5)	5477	38.1 (36.7–39.5)	2156	38.3 (36.7–40.0)
1–3 days/week	8427	46.5 (45.4–47.6)	7953	46.6 (45.5–47.7)	457	42.2 (38.8–45.7)	6191	48.2 (46.9-49.4)	2233	39.5 (38.0-41.0)
0 days/week	3015	15.3 (14.6-16.1)	2808	15.2 (14.5-16.0)	199	18.6 (15.9-21.7)	1773	13.7 (12.8-14.6)	1239	22.2 (20.9-23.4)
Body mass index *										
<18.5 kg/m^2^	528	2.2 (2.0-2.5)	494	2.2 (2.0-2.4)	32	2.6 (1.7-3.9)	381	2.2 (1.9-2.5)	146	2.4 (2.0-2.8)
18.5–24.9 kg/m^2^	6975	33.5 (32.4–34.6)	6559	33.4 (32.4–34.6)	409	35.1 (31.7–38.7)	5086	33.6 (32.3–34.8)	1888	33.2 (31.8–34.6)
25.0–29.9 kg/m^2^	5469	31.9 (30.8–33.1)	5183	32.2 (31.0–33.3)	273	26.1 (23.3–29.0)	3828	32.0 (30.7–33.4)	1638	31.5 (30.1–33.0)
≥30 kg/m^2^	5636	32.4 (31.2–33.5)	5259	32.2 (31.0–33.4)	367	36.2 (32.8–39.8)	3810	32.2 (30.9–33.6)	1824	32.9 (31.3–34.4)
Heavy alcohol use *†										
No	17,920	95.3 (94.8–95.7)	16,901	95.5 (95.0–95.9)	990	90.9 (88.8–92.6)	12,922	96.8 (96.4–97.2)	4988	88.8 (87.7–89.9)
Yes	1111	4.7 (4.3–5.2)	1008	4.5 (4.1–5.0)	98	9.1 (7.4–11.2)	510	3.2 (2.8–3.6)	600	11.2 (10.1–12.3)
**Insurance status †**										
Insured	15,495	85.0 (84.2–85.8)	14,587	85.1 (84.2–85.9)	889	83.0 (80.2–85.5)	11230	87.2 (86.3–88.1)	4260	75.9 (74.3–77.4)
Uninsured	3452	15.0 (14.2–15.8)	3240	14.9 (14.1–15.8)	199	17.0 (14.5–19.8)	2100	12.8 (11.9–13.7)	1345	24.1 (22.6–25.7)
Marital status *†										
Married	6393	49.9 (48.8–51.1)	6034	50.5 (49.3–51.6)	346	34.6 (31.4–38.0)	4561	53.4 (52.1–54.6)	1830	35.6 (33.6–37.6)
Widowed, divorced or separated	2378	13.4 (12.7–14.2)	2196	13.2 (12.4–14.1)	176	18.9 (16.4–21.7)	1086	10.8 (10.0-11.6)	1289	24.6 (23.1-26.1)
Never married	10,150	36.6 (35.7–37.6)	9570	36.3 (35.3-37.3)	566	46.5 (43.0-50.0)	7692	35.9 (34.8-37.0)	2451	39.8 (37.9-41.8)
**Hyperlipidemia †**										
No	16,867	84.3 (83.4-85.1)	15,891	84.2 (83.4-85.0)	947	85.3 (82.9-87.5)	12,089	84.5 (83.6-85.5)	4768	83.1 (82.0-84.1)
Yes	2277	15.7 (14.9–16.6)	2120	15.8 (15.0–16.6)	152	14.7 (12.5–17.1)	1390	15.5 (14.5–16.4)	885	16.9 (15.9–18.0)
**Diabetes mellitus**										
No	17,784	91.5 (90.8–92.1)	16,750	91.5 (90.8–92.2)	1005	91.1 (88.7–93.0)	12,655	91.6 (90.8–92.4)	5119	90.9 (90.0–91.7)
Yes	1336	8.5 (7.9–9.2)	1240	8.5 (7.8–9.2)	91	8.9 (7.0–11.3)	815	8.4 (7.6–9.2)	519	9.1 (8.3–10.0)

Reported statistics (other than frequencies) represent weighted values according to PATH Study specifications. Due to some missing data points, subgroup frequencies do not all add up to the full analytic sample (n = 19,147). Details provided in supplement file. * Indicates Rao-Scott χ^2^ test *p* < 0.05 comparing current vaper v. never/former vaper. † Indicates Rao-Scott χ^2^ test *p* < 0.05 comparing current smoker v. never/former smoker. CI = confidence interval.

**Table 2:** Prevalence of hypertension and multivariable odds for hypertension among the analytic sample, modeling current smoking and vaping as separate variables.

Variable		Prevalence of Hypertension		Multivariable Odds of Hypertension
	n	Cases	% (95% CI)	aOR (95% CI)
Current vaper				
No	18,013	2650	17.1 (16.3–18.0)	REF
Yes	1100	201	21.3 (18.7–24.2)	1.31 (1.05–1.63)
Current smoker				
No	13,481	1618	16.0 (15.1–17.0)	REF
Yes	5654	1241	22.6 (21.2–24.0)	1.27 (1.10–1.47)
Age				
18–24 years	7238	310	4.5 (3.8–5.1)	REF
25–34 years	4985	573	10.9 (9.7–12.2)	2.33 (1.82–2.99)
35–44 years	3549	798	18.3 (16.8–19.9)	3.58 (2.82–4.55)
45–54 years	3375	1178	31.1 (29.2–33.0)	6.19 (4.90–7.83)
Sex				
Female	10,505	1465	15.2 (14.1–16.4)	REF
Male	8626	1393	19.7 (18.4–20.9)	1.60 (1.39–1.85)
Race-ethnicity				
Non-Hispanic White	10,428	1538	17.6 (16.4–18.8)	REF
Non-Hispanic Black	2674	581	26.1 (24.0–28.4)	1.56 (1.32–1.84)
Hispanic	4256	452	12.8 (11.4–14.3)	0.67 (0.54–0.82)
Non-Hispanic Other	1580	230	12.4 (10.1–15.0)	0.95 (0.72–1.24)
Annual household income				
≥USD 50,000	6793	927	15.7 (14.3–17.3)	REF
<USD 50,000	11,045	1801	19.2 (18.1–20.5)	1.32 (1.09–1.60)
Education status				
Bachelors and beyond	4085	557	13.9 (12.4–15.7)	REF
Some college	6812	1069	18.1 (16.9–19.5)	1.10 (0.92–1.32)
High school or less	8166	1223	19.3 (18.1–20.5)	1.08 (0.89–1.33)
Insurance status				
Insured	15,495	2437	18.0 (17.0–19.0)	REF
Uninsured	3452	400	13.4 (12.0–15.0)	0.70 (0.58–0.85)
Marital status				
Married	6393	1208	18.0 (16.8–19.3)	REF
Widowed, divorced or separated	2378	648	26.0 (23.6–28.6)	1.26 (1.01–1.57)
Never married	10,150	960	12.9 (11.9–13.9)	1.23 (1.04–1.45)
Leisure-time physical activity				
≥4 days/week	7638	953	15.3 (14.1–16.5)	REF
1–3 days/week	8427	1244	16.5 (15.5–17.6)	0.92 (0.80–1.07)
0 days/week	3015	651	24.3 (22.2–26.5)	1.18 (0.98–1.42)
Body mass index				
<18.5 kg/m^2^	528	26	5.9 (3.3–10.2)	REF
18.5–24.9 kg/m^2^	6975	420	6.6 (5.8–7.5)	1.10 (0.52–2.34)
25.0–29.9 kg/m^2^	5469	790	15.4 (14.2–16.7)	1.98 (0.95–4.13)
≥30 kg/m^2^	5636	1527	30.5 (28.9–32.2)	4.11 (1.98–8.55)
Heavy alcohol use				
No	17,920	2629	17.0 (16.2–17.9)	REF
Yes	1111	211	21.4 (18.4–24.8)	1.33 (1.01–1.75)
Hypercholesterolemia				
No	16,867	1797	12.5 (11.8–13.3)	REF
Yes	2277	1062	42.6 (39.3–45.9)	2.85 (2.36–3.45)
Diabetes mellitus				
No	17,784	2163	13.9 (13.1–14.8)	REF
Yes	1336	683	52.2 (47.9–56.4)	2.95 (2.39–3.65)

Reported statistics (other than frequencies) represent weighted values according to PATH Study specifications. Due to missing data points, subgroup frequencies do not all add up to the full analytic sample (n = 19,147). Details provided in supplement file. aOR = adjusted odds ratio; CI = confidence interval.

## Data Availability

Publicly available datasets were analyzed in this study. This data can be found here: [https://www.icpsr.umich.edu/icpsrweb/NAHDAP/search/studies?q=PATH (accessed on 8 March 2021)].

## Supplementary Materials

The following are available online at https://www.mdpi.com/2305-6304/9/3/52/s1, Figure S1: Flow diagram describing the analytic sample and detailing the study’s case definition for hypertension, Figure S2: Multivariable odds for hypertension among PATH Wave 3 respondents aged 18–54 years including users of ‘other’ tobacco products, modeling smoking and vaping as a composite variable, Table S1: Descriptive statistics of the analytic sample, according to a composite variable accounting for current vaping status, current smoking status, and former smoking status, Table S2: Missing data in the analytic sample, Table S3: Respondents excluded from regression analyses due to missing data points during primary analyses of the analytic sample, Table S4: Prevalence of hypertension and multivariable odds for hypertension among Wave 3 PATH respondents, stratified by age.
