# Supplementary material for: Cross-Sectional Associations of Smoking and E-cigarette Use with Self-Reported Diagnosed Hypertension: Findings from Wave 3 of the Population Assessment of Tobacco and Health Study
Source: Toxics. 2021 Mar 9;9(3):52. doi: 10.3390/toxics9030052 (PMC7999635; doi:10.3390/toxics9030052)
Supplement: Supplementary file 1 [file toxics-09-00052-s001.pdf]

toxics

Supplementary Materials: Cross-Sectional Associations of Smoking and E-cigarette Use with Self-Reported Diagnosed Hypertension: Findings from Wave 3 of the Population Assessment of Tobacco and Health Study

Connor R. Miller, Hangchuan Shi, Dongmei Li and Maciej L. Goniewicz

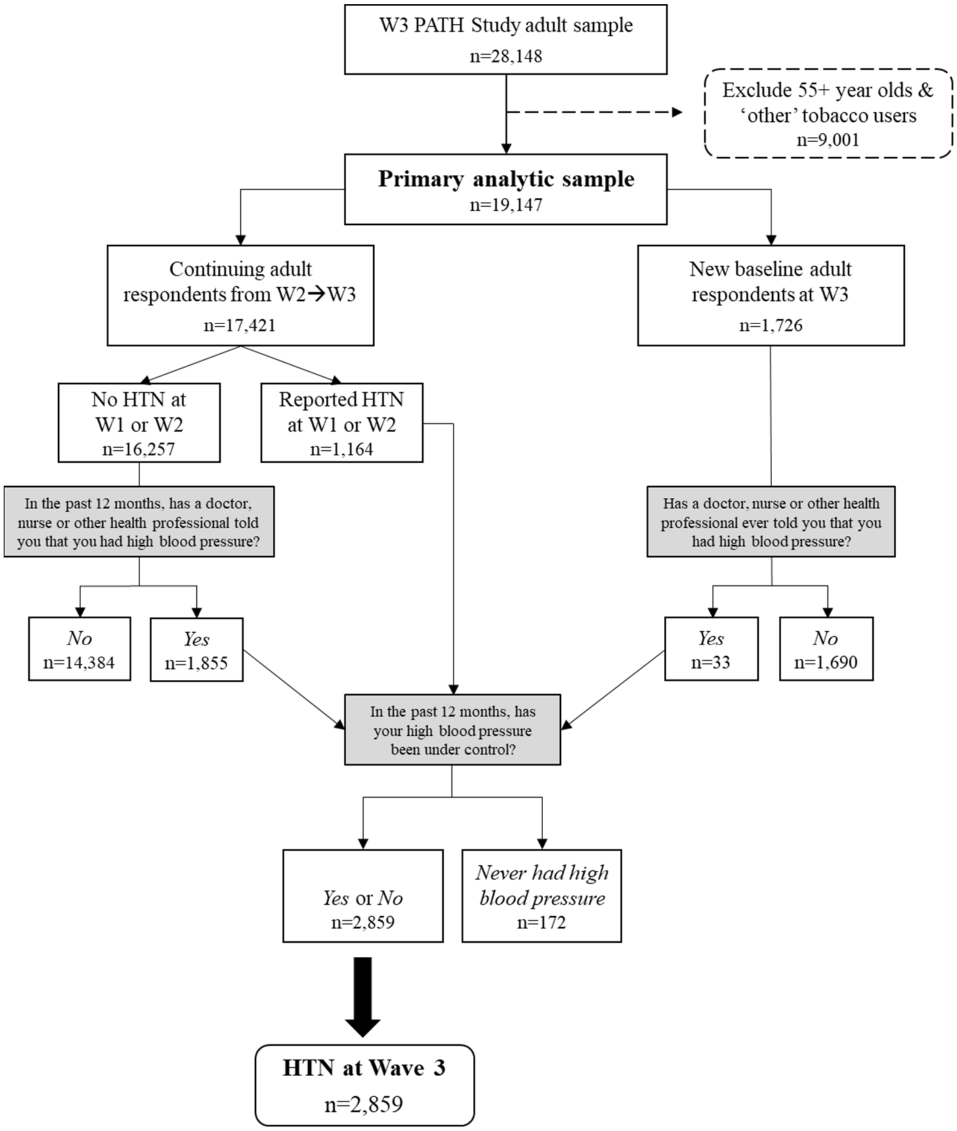

Supplemental Figure S1. Flow diagram describing the analytic sample and detailing the study's case definition for hypertension.

HTN=Hypertension; W=Wave.

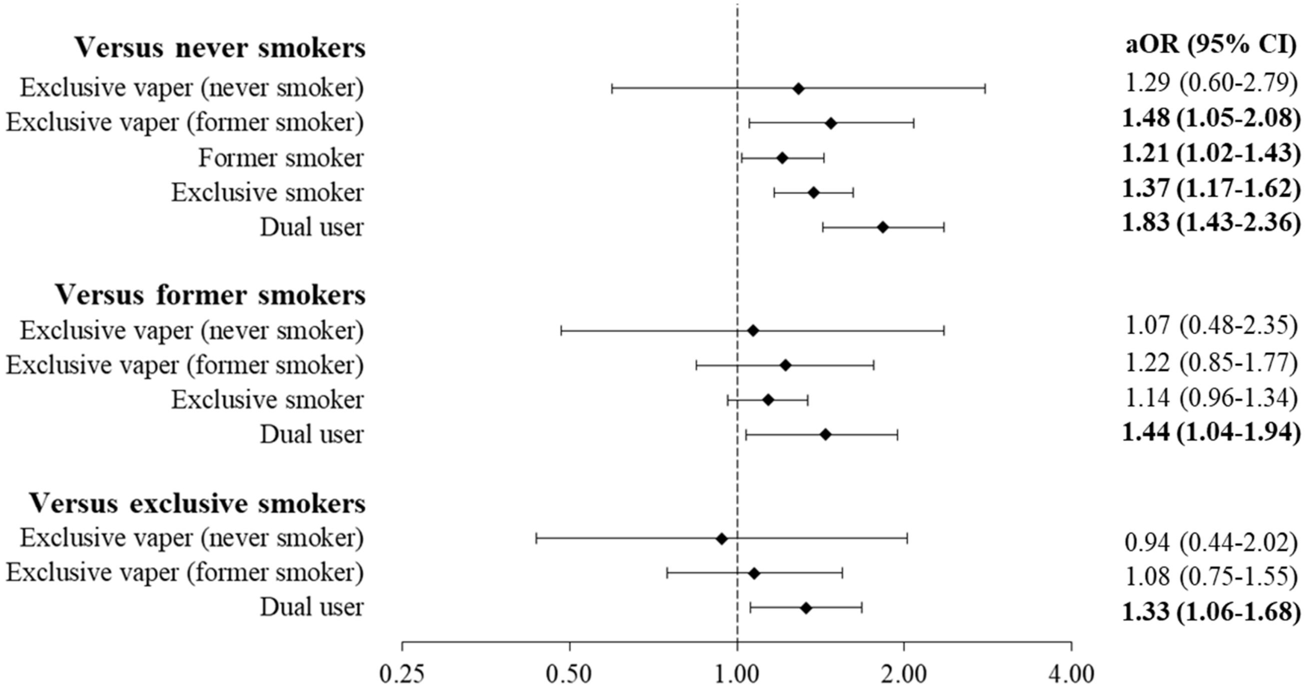

Supplemental Figure S2. Multivariable odds for hypertension among PATH Wave 3 respondents aged 18-54 years **including users of 'other' tobacco users**, modeling smoking and vaping as a composite variable.

Note: all results come from weighted logistic regression models controlling for age, race-ethnicity, sex, annual household income, education, leisure time physical activity, BMI, heavy alcohol use, insurance status, marital status, hypercholesterolemia, diabetes mellitus, current-established 'other' combusted tobacco use, and current-established 'other' smokeless tobacco use. aOR=adjusted odds ratio; CI=confidence interval.

Table S1. Descriptive statistics of the analytic sample, according to a composite variable accounting for current vaping status, current smoking status, and former smoking status.

	**Never Smoker (n=8783)**		**Current Exclusive Vaper: Never Smoker (n=183)**		**Current Exclusive Vaper: Former Smoker (n=334)**		**Former Smoker (n=3938)**		**Current Exclusive Smoker (n=5056)**		**Current Dual User (n=581)**	
	**n**	**% (95% CI)**	**n**	**% (95% CI)**	**n**	**% (95% CI)**	**n**	**% (95% CI)**	**n**	**% (95% CI)**	**n**	% (95% CI)
**Hypertension**												
No	7954	86.4 (85.2-87.4)	171	92.4 (85.9-96.1)	270	77.5 (70.8-83.0)	3232	79.1 (77.2-80.9)	3941	77.6 (76.1-79.1)	454	76.2 (72.4-79.6)
Yes	823	13.6 (12.6-14.8)	10	7.6 (3.9-14.1)	64	22.5 (17.0-29.2)	702	20.9 (19.1-22.8)	1106	22.4 (20.9-23.9)	127	23.8 (20.4-27.6)
**Age**												
18-24 years	4860	24.9 (24.0-25.8)	156	78.7 (69.6-85.7)	71	15.0 (11.6-19.3)	907	9.2 (8.3-10.1)	918	11.9 (10.9-12.9)	161	18.7 (15.6-22.3)
25-34 years	1988	28.5 (27.0-30.1)	21	14.6 (9.1-22.6)	99	33.7 (28.0-39.8)	1117	22.4 (21.0-23.9)	1552	30.5 (28.8-32.3)	161	30.1 (26.3-34.3)
35-44 years	1044	23.5 (22.0-25.0)	4	4.3 (1.5-11.3)	92	26.9 (22.4-32.0)	972	30.4 (28.4-32.6)	1263	28.2 (26.6-29.9)	143	28.5 (24.6-32.7)
45-54 years	891	23.1 (21.8-24.5)	2	2.4 (0.6-8.6)	72	24.4 (19.2-30.5)	942	38.0 (35.8-40.2)	1323	29.4 (27.9-30.9)	116	22.7 (19.1-26.6)
**Sex**												
Female	4919	56.3 (55.0-57.5)	76	40.4 (32.0-49.5)	152	40.7 (34.0-47.7)	2131	52.7 (50.9-54.5)	2767	49.9 (48.3-51.4)	310	48.5 (44.4-52.7)
Male	3852	43.7 (42.5-45.0)	107	59.6 (50.5-68.0)	182	59.3 (52.3-66.0)	1805	47.3 (45.5-49.1)	2287	50.1 (48.6-51.7)	271	51.5 (47.3-55.6)
**Race-ethnicity**												
Non-Hispanic White	4143	53.7 (52.3-55.1)	99	55.5 (46.6-64.1)	252	77.8 (72.4-82.4)	2209	64.5 (62.7-66.3)	3193	67.5 (65.7-69.2)	433	79.6 (75.3-83.4)
Non-Hispanic Black	1410	12.8 (12.0-13.6)	13	9.0 (4.8-16.1)	18	5.1 (3.0-8.7)	475	9.1 (8.1-10.1)	674	12.5 (11.4-13.8)	26	4.6 (3.0-6.9)
Hispanic	2363	22.3 (21.4-23.3)	56	26.9 (19.7-35.6)	36	9.8 (7.0-13.5)	903	19.0 (17.6-20.4)	746	14.1 (13.0-15.2)	69	9.0 (6.6-12.0)
Non-Hispanic Other	785	11.2 (10.2-12.2)	14	8.6 (4.3-16.4)	28	7.3 (4.7-11.2)	310	7.5 (6.4-8.7)	364	5.9 (5.2-6.8)	52	6.8 (4.7-9.8)
**Annual household income**												
≥ $50000	3376	50.2 (48.6-51.7)	47	31.6 (23.8-40.7)	136	45.9 (40.0-51.9)	1779	59.5 (57.2-61.7)	1232	29.4 (27.5-31.3)	156	33.0 (28.4-37.8)
< $50000	4665	49.8 (48.3-51.4)	116	68.4 (59.3-76.2)	182	54.1 (48.1-60.0)	1937	40.5 (38.3-42.8)	3588	70.6 (68.7-72.5)	390	67.0 (62.2-71.6)
**Education status**												
Bachelors & beyond	2200	36.2 (35.0-37.3)	14	10.5 (5.9-18.1)	52	15.9 (11.8-21.1)	1221	35.9 (34.1-37.8)	502	11.6 (10.3-13.0)	67	14.4 (10.8-19.0)
Some college	3034	31.4 (30.2-32.6)	64	37.5 (29.4-46.4)	168	51.0 (45.3-56.8)	1449	33.8 (31.8-35.8)	1752	34.4 (32.7-36.1)	263	43.9 (39.2-48.8)
High school or less	3511	32.5 (31.3-33.7)	105	52.0 (42.8-61.0)	113	33.0 (26.8-40.0)	1258	30.3 (28.4-32.2)	2780	54.0 (52.2-55.8)	244	41.6 (36.6-46.8)

**Leisure-time physical activity**												
≥ 4 days/week	3634	38.6 (37.1-40.3)	82	44.4 (36.4-52.8)	125	39.1 (33.0-45.5)	1530	36.7 (34.5-39.1)	1920	38.4 (36.8-40.1)	230	37.8 (33.4-42.4)
1-3 days/week	4057	48.3 (46.8-49.8)	84	48.4 (40.0-56.8)	143	41.1 (35.7-46.7)	1800	48.3 (46.1-50.5)	1998	39.3 (37.8-40.8)	230	41.6 (36.7-46.6)
0 days/week	1067	13.1 (11.9-14.3)	17	7.2 (4.3-11.9)	66	19.8 (15.3-25.2)	593	15.0 (13.6-16.4)	1118	22.3 (21.1-23.6)	116	20.6 (16.7-25.1)
**Body mass index**												
< 18.5 kg/m^2	300	2.6 (2.2-3.0)	4	1.6 (0.6-4.8)	13	3.5 (1.9-6.3)	60	1.3 (0.9-1.8)	131	2.4 (2.0-3.0)	15	2.3 (1.3-4.1)
18.5-24.9 kg/m^2	3555	35.2 (33.7-36.7)	85	45.9 (37.4-54.7)	105	30.4 (24.8-36.6)	1234	30.0 (28.1-32.0)	1665	32.9 (31.5-34.4)	218	35.5 (31.1-40.2)
25.0-29.9 kg/m^2	2412	31.6 (30.1-33.2)	52	29.9 (23.7-37.0)	84	24.6 (19.7-30.3)	1217	33.5 (31.4-35.6)	1497	32.2 (30.7-33.7)	136	26.1 (21.9-30.8)
≥ 30 kg/m^2	2263	30.7 (29.1-32.3)	38	22.5 (15.8-31.0)	128	41.6 (34.7-48.8)	1320	35.2 (33.0-37.5)	1617	32.5 (30.9-34.1)	201	36.1 (32.1-40.3)
**Heavy alcohol use**												
No	8471	97.5 (97.1-97.9)	158	80.6 (71.9-87.1)	312	93.5 (90.1-95.8)	3746	95.9 (95.0-96.6)	4455	88.5 (87.3-89.6)	518	91.7 (89.1-93.7)
Yes	282	2.5 (2.1-2.9)	23	19.4 (12.9-28.1)	21	6.5 (4.2-9.9)	177	4.1 (3.4-5.0)	544	11.5 (10.4-12.7)	54	8.3 (6.3-10.9)
**Insurance status**												
Insured	7296	86.3 (85.2-87.3)	147	82.8 (75.5-88.3)	276	83.6 (77.6-88.2)	3325	89.7 (88.4-90.9)	3786	75.1 (73.4-76.8)	465	82.8 (79.3-85.7)
Uninsured	1378	13.7 (12.7-14.8)	32	17.2 (11.7-24.5)	56	16.4 (11.8-22.4)	584	10.3 (9.1-11.6)	1228	24.9 (23.2-26.6)	110	17.2 (14.3-20.7)
**Marital status**												
Married	2477	49.9 (48.2-51.6)	19	10.6 (6.7-16.5)	142	43.0 (36.8-49.4)	1869	62.4 (60.6-64.3)	1638	35.6 (33.6-37.6)	185	35.3 (30.6-40.2)
Widowed divorced or separated	518	9.5 (8.6-10.6)	8	6.6 (3.1-13.5)	53	17.9 (13.8-22.8)	490	12.9 (11.8-14.2)	1171	24.9 (23.3-26.5)	115	22.5 (18.7-26.8)
Never married	5712	40.5 (39.0-42.1)	156	82.7 (74.9-88.5)	136	39.1 (32.4-46.3)	1520	24.6 (23.1-26.2)	2173	39.6 (37.7-41.5)	272	42.3 (37.7-47.0)
**Hyperlipidemia**												
No	8061	86.8 (85.5-87.9)	181	99.1 (96.5-99.8)	273	81.7 (76.7-85.8)	3341	79.6 (77.7-81.3)	4265	83.0 (81.8-84.1)	491	84.3 (80.8-87.2)
Yes	721	13.2 (12.1-14.5)	2	0.9 (0.2-3.5)	60	18.3 (14.2-23.3)	597	20.4 (18.7-22.3)	790	17.0 (15.9-18.2)	90	15.7 (12.8-19.2)
**Diabetes mellitus**												
No	8346	92.4 (91.3-93.3)	174	95.8 (91.2-98.1)	301	89.2 (83.5-93.1)	3599	90.0 (88.5-91.4)	4579	90.9 (90.0-91.7)	528	91.1 (88.3-93.3)
Yes	433	7.6 (6.7-8.7)	8	4.2 (1.9-8.8)	32	10.8 (6.9-16.5)	335	10.0 (8.6-11.5)	463	9.1 (8.3-10.0)	51	8.9 (6.7-11.7)

Reported statistics (other than frequencies) represent weighted values according to PATH Study specifications.
Due to some missing data points, subgroup frequencies do not all add up to the full analytic sample (n=19,147). Details provided in supplement file.
CI=confidence interval.

Table S2. Missing data in the analytic sample.

Variable	Analytic Sample	n Missing	Unweighted % of Analytic Sample Missing
**Exposure(s)**			
Vaping (2 cat. separate)	19,147	34	0.2%
Smoking (2 cat. separate)	19,147	12	0.1%
Vaping & smoking (6 cat. composite)	19,147	272	1.4%
**Outcome**			
Hypertension	19,147	21	0.1%
**Covariates**			
Age	19,147	0	0.0%
Sex	19,147	16	0.1%
Race-ethnicity	19,147	209	1.1%
Annual household income	19,147	1309	6.8%
Education status	19,147	84	0.4%
Leisure-time physical activity	19,147	67	0.3%
Body mass index	19,147	539	2.8%
Heavy alcohol use	19,147	116	0.6%
Insurance status	19,147	200	1.0%
Marital status	19,147	226	1.2%
Hypercholesterolemia	19,147	3	0.0%
Diabetes mellitus	19,147	27	0.1%

Table S3. Respondents excluded from regression analyses due to missing data points during primary analyses of the analytic sample.

Regression Model	Analytic Sample	n Used in Model	Case-Wise Deletions	Unweighted % of Analytic Sample Excluded
Vaping & smoking (2 cat. separate)	19,147	16,879	2268	11.8%
Vaping & smoking (6 cat. composite)	19,147	16,699	2448	12.8%

Table S4. Prevalence of hypertension and multivariable odds for hypertension among Wave 3 PATH respondents, stratified by age.

Tobacco Use	n	HTN Cases, n (%)	aOR (95% CI)
**18-34 years** *(n=13,677)*			
**Smoking**			
Never-established smoker	5467	262 (6.9)	1.00 (ref)
Former-established smoker	3889	331 (9.3)	**1.38 (1.06-1.81)**
Current-established smoker	4321	511 (12.0)	**1.51 (1.18-1.93)**
**Vaping**			
Never-established vaper	9541	670 (8.1)	1.00 (ref)
Former-established vaper	3116	313 (10.9)	1.26 (0.99-1.61)
Current-established vaper	1020	121 (12.7)	1.25 (0.94-1.66)
**35-54 years** *(n=7,525)*			
**Smoking**			
Never-established smoker	1609	380 (20.5)	1.00 (ref)
Former-established smoker	2581	740 (27.1)	**1.33 (1.06-1.66)**
Current-established smoker	3335	1113 (31.8)	**1.31 (1.07-1.60)**
**Vaping**			
Never-established vaper	5505	1565 (24.6)	1.00 (ref)
Former-established vaper	1513	502 (31.6)	1.12 (0.84-1.48)
Current-established vaper	507	166 (34.0)	1.18 (0.86-1.62)
**55+ years** *(n=5,857)*			
**Smoking**			
Never-established smoker	1209	662 (54.6)	1.00 (ref)
Former-established smoker	2489	1437 (57.6)	1.02 (0.83-1.25)
Current-established smoker	2159	1202 (55.1)	0.94 (0.76-1.15)
**Vaping**			
Never-established vaper	4823	2729 (56.2)	1.00 (ref)
Former-established vaper	813	462 (57.7)	0.91 (0.65-1.28)
Current-established vaper	221	110 (48.1)	**0.66 (0.49-0.88)**

In addition to separate smoking and vaping status variables, models were adjusted for age, race-ethnicity, sex, annual household income, education, leisure time physical activity, BMI, heavy alcohol use, insurance status, marital status, hypercholesterolemia, diabetes mellitus, current-established 'other' combusted tobacco use, and current-established 'other' smokeless tobacco use.
Data presented for 27,059 Wave 3 PATH respondents with available data for age, current and former smoker, and current smoking status, former smoking status, current vaping status and former vaping status (n missing / excluded = 1,089).
HTN=hypertension, aOR=adjusted odds ratio, CI=confidence interval.

Table S5. Prevalence of annual doctor visits during the past three years among the analytic sample, stratified by tobacco use.

	Overall	Visited Doctor in P12M: Yes		
	n	n	% (95%CI)	Rao-Scott χ^2 p value
**Response at Wave 1** (n=15,906*)				
Never smoker	7435	5769	77.7 (76.4-79.0)	<0.001
Exclusive vaper (never smoker)	81	61	76.3 (65.9-84.2)	
Exclusive vaper (former smoker)	318	230	70.2 (63.7-76.0)	
Former smoker	2489	1926	79.0 (76.8-81.1)	
Exclusive smoker	4883	3424	68.7 (66.9-70.3)	
Dual user	534	409	75.1 (69.8-79.7)	
**Response at Wave 2** (n=16,829†)				
Never smoker	8577	5759	68.7 (67.2-70.1)	<0.001
Exclusive vaper (never smoker)	123	81	62.3 (52.7-71.0)	
Exclusive vaper (former smoker)	316	205	61.7 (54.7-68.2)	
Former smoker	2436	1696	72.6 (70.0-75.1)	
Exclusive smoker	4797	2914	59.5 (57.8-61.3)	
Dual user	539	364	69.1 (64.9-72.9)	
**Response at Wave 3** (n=19,147)				
Never smoker	10,164	7101	72.3 (70.9-73.6)	<0.001
Exclusive vaper (never smoker)	183	122	63.1 (53.6-71.7)	
Exclusive vaper (former smoker)	334	225	66.9 (61.0-72.2)	
Former smoker	2,557	1849	76.2 (74.2-78.0)	
Exclusive smoker	5,056	3145	61.5 (59.7-63.3)	
Dual user	581	404	70.0 (65.3-74.3)	

Reported statistics (other than frequencies) represent weighted values according to PATH Study Wave 3 specifications.
*excluding n=3241 respondents who were in the youth cohort during Wave 1 of the PATH Study (youth were not asked about annual doctor visits).
†excluding n=2318 respondents who were in the youth cohort during Wave 2 of the PATH Study (youth were not asked about annual doctor visits).
P12M=Past 12 months.
